# A Pilot Study on the Short‐Term Effects of an Electric Knee–Ankle–Foot Orthosis on Gait Performance and Physiological Cost Index in Patients With Hemiplegia: Influence of Initial Balance Ability Assessed by the Berg Balance Scale

**DOI:** 10.1155/bmri/5528235

**Published:** 2026-04-06

**Authors:** Hyuk-Jae Choi, Yoon Heo, Jong-Won Lee, Hyeonseok Cho, Ju-Hwan Bae, In Ho Hwang, Mi Jung Kim, Chang-Yong Ko

**Affiliations:** ^1^ Rehabilitation Engineering Research Institute, Korea Workers’ Compensation & Welfare Service, Incheon, Republic of Korea; ^2^ Department of Physical Medicine and Rehabilitation, Hanyang University Medical Center, Seoul, Republic of Korea, hyumc.com; ^3^ Refind Inc, Wonju, Republic of Korea

**Keywords:** Berg Balance Scale, electric knee–ankle–foot orthosis, gait performance, hemiplegia, physiological cost index, stroke rehabilitation

## Abstract

Gait impairment is one of the most disabling consequences of stroke and substantially limits functional independence. While traditional orthoses provide joint stabilization, they often restrict natural motion and lack adaptive support. This study is aimed at evaluating the short‐term effects of an electric knee–ankle–foot orthosis (E‐KAFO) on gait performance and energy efficiency in patients with hemiplegia and at assessing how baseline balance ability, measured using the Berg Balance Scale (BBS), influences outcomes. This pre–post intervention, nonrandomized study enrolled poststroke survivors (*n* = 22), who were stratified into high‐ (BBS > 45) and low‐ (BBS ≤ 45) balance groups (HBG and LBG, respectively). Participants underwent gait assessments under two conditions: body‐powered condition (BP_C), to evaluate rehabilitation effects independently of the device, and E‐KAFO_C, to assess short‐term assistive efficacy immediately after device use. All assessments were conducted before and after a 3‐week intervention comprising seven E‐KAFO training sessions. The primary outcome was gait speed, measured using the 5‐m walk test (5‐mWT). The 3‐min and 6‐min walk tests (3‐MWT and 6‐MWT) and the physiological cost index (PCI) were also evaluated to assess gait endurance and energy efficiency. The 5‐mWT and 3‐MWT/6‐MWT were performed in randomized order to minimize order‐related bias. No standard rehabilitation‐only control group was included. Assistive effects (E‐KAFO_C) and rehabilitative effects (BP_C) were analyzed and reported separately. Two‐way repeated measures analysis of variance was used to assess the effects of group, time, and their interaction. Significant improvements in gait speed and endurance were observed across both conditions, with the LBG showing greater relative gains postintervention. In the E‐KAFO_C condition, the LBG demonstrated a 33.31% increase in 5‐mWT speed and a 25.51% increase in 3‐MWT distance. Notably, PCI decreased in both groups, although the reduction was significant only in the LBG. These findings underscore the distinction between the short‐term assistive benefits of the E‐KAFO and the rehabilitative improvements captured by BP_C outcomes, emphasizing that BP_C results serve as the primary indicator of rehabilitation effectiveness. Importantly, baseline balance ability assessed by the BBS influenced assistive and rehabilitative responses, supporting its relevance for patient stratification. Given the exploratory design and limited sample size, these findings are hypothesis‐generating and require confirmation in larger randomized controlled trials.

**Trial Registration:**

WHO International Clinical Trials Registry Platform, supported by the Korea Disease Control and Prevention Agency (KDCA): KCT0011412

## 1. Introduction

Gait impairment is one of the most severe sequelae of stroke, limiting mobility and independence [1, 2]. Approximately one‐third of survivors remain unable to walk independently after 6 months [3], and in the long term, only about 60% regain independent walking ability [4]. Such impairments increase fall risk and caregiver burden, making gait recovery a primary rehabilitation goal closely tied to quality of life [5, 6].

Traditional rehabilitation focuses on restoring gait patterns through balance, strengthening, coordination, and treadmill training with body weight support, which has proven effective [7–9]. These approaches are often complemented by orthoses such as ankle–foot orthoses (AFOs) and knee–ankle–foot orthoses (KAFOs), which stabilize limbs and improve gait speed and stride length. AFOs reduce foot drop and aid foot clearance [10], while KAFOs stabilize the knee in cases of quadriceps weakness or hyperextension [11].

Although traditional body‐powered orthoses (passive braces) can improve gait safety and certain gait parameters, they have limitations. Rigid AFOs or KAFOs (typically made of hard plastic or metal) may restrict the natural range of motion and reduce sensory feedback, potentially leading to inefficient or unnatural gait patterns [12]. Moreover, despite wearing orthoses, patients often resort to compensatory strategies such as excessive hip hiking and circumduction gait during walking [12–14]. Because these passive devices only support or restrict joint movement, they cannot fully address the neuromuscular deficits that follow stroke [15, 16].

Recently, wearable powered exoskeletons have emerged as an alternative [17, 18]. Among these, the electric knee–ankle–foot orthosis (E‐KAFO) uses knee actuators and inertial sensors to assist movement in real time, providing controlled knee flexion–extension and adaptive support aligned with user intent [19–21]. By enhancing gait efficiency, symmetry, and natural motion, it differs fundamentally from passive braces.

Powered orthoses have been shown to increase walking speed and step length while reducing muscular workload and overall energy expenditure in individuals with paralysis [2, 22–24]. However, evidence remains limited regarding which stroke patients benefit most from E‐KAFO use.

This study evaluated the short‐term effects of an E‐KAFO on gait performance and physiological effort in stroke survivors, with particular attention to whether baseline balance ability modifies these effects. Participants were divided into high‐balance group (HBG) and low‐balance group (LBG) according to the Berg Balance Scale (BBS). We hypothesized that, compared with passive orthoses, the E‐KAFO would produce greater improvements in gait performance (speed and endurance) and reduce energy cost, with more pronounced effects in the LBG. This hypothesis is supported by previous findings that BBS scores at admission predict walking recovery and community ambulation after stroke and that baseline BBS predicts responsiveness to robot‐assisted gait training [25, 26]. These associations highlight balance capacity as a key determinant of gait outcomes and intervention responsiveness, suggesting that E‐KAFO use may yield clinically meaningful benefits for patients with impaired balance.

## 2. Materials and Methods

### 2.1. Participants

This study included 22 patients with poststroke hemiplegia who were able to use the E‐KAFO. The objective was to evaluate the short‐term assistive effects of the E‐KAFO on gait performance and energy efficiency (E‐KAFO condition [E‐KAFO_C]), independent of its rehabilitative effectiveness (body‐powered condition [BP_C]). To minimize adaptation bias, each participant completed a brief familiarization session prior to the E‐KAFO_C assessment. The inclusion criteria were as follows: age ≥ 18 years, presence of gait impairment with the ability to walk at least 5 m with assistance or an assistive device, and adequate cognitive function, as indicated by a Mini‐Cog score ≥ 3 [27, 28]. Permission to use the Mini‐Cog was granted by Dr. Soo Borson, its codeveloper, via official email correspondence.

Exclusion criteria included severe spasticity (Modified Ashworth Scale [MAS] ≥ 2 at the ankle or knee), cardiovascular or respiratory disorders limiting gait performance, use of a cardiac pacemaker, or any other orthopedic or neurodegenerative condition affecting walking ability.

Participants were stratified into two groups based on their BBS score at baseline: a HBG (BBS > 45, *n* = 11) and a LBG (BBS ≤ 45, *n* = 11) [29, 30]. All participants provided written informed consent after receiving a detailed explanation of the study’s purpose and procedures and prior to enrollment in the study. This study was conducted in accordance with the ethical principles of the Declaration of Helsinki and was approved by the Hanyang University Institutional Review Board committee (Approval No. HYUIRB‐202304‐025‐4). In accordance with the World Health Organization (WHO) definition of a clinical trial, this study was retrospectively registered through the Clinical Research Information Service (CRiS), Republic of Korea, a WHO‐recognized primary registry, and is accessible via the WHO International Clinical Trials Registry Platform (ICTRP).

Baseline and clinical characteristics are summarized in Table 1, including mean age (57.73 ± 10.32 years), sex distribution (19 men and 3 women), height (169.38 ± 7.13 cm), weight (70.84 ± 12.45 kg), and poststroke duration. The mean poststroke duration did not differ significantly between groups (HBG: 66.36 ± 47.17 months; LBG: 45.64 ± 47.31 months; *p* = 0.316) (Table 1).

**Table 1:** Characteristics of hemiplegia subjects (*n* = 22).

Variable	HBG (*n* = 11)	LBG (*n* = 11)	*p* values
Gender			
Male	9	10	
Female	2	1	
Age (year)	57.45 ± 12.02^a^	58.00 ± 8.88	0.905
Height (cm)	167.77 ± 6.44	170.60 ± 7.32	0.302
Weight (kg)	68.65 ± 11.34	73.02 ± 13.66	0.424
BMI (kg/cm^2^)	24.29 ± 3.06	24.76 ± 2.92	0.715
Injury dur. (mo.)	66.36 ± 47.17	45.64 ± 47.31	0.316
Hemiplegic side			
Right	7	6	
Left	4	5	
CVA type			
Inf.	10	4	
Hemo.	1	5	
Etc.	0	2	
mFAC	4.45 ± 0.52	3.91 ± 0.54	0.080
Major ambulation			
W/C	0	1	
Cane	3	7	
Independent gait	8	3	
TUG (s)	13.47 ± 3.22	23.98 ± 5.93	< 0.001
MAS of affected side			
Hip Fle./Ext.	0.09 ± 0.30	0.55 ± 0.65	
Knee Fle./Ext.	0.27 ± 0.47	0.82 ± 0.56	
Ankle D.Fle./P.Fle.	0.27 ± 0.47	0.18 ± 0.40	
BBS	51.00 ± 3.97	37.82 ± 5.46	< 0.001
MMT of affected side			
Hip Fle./Ext.	3.55 ± 0.52	2.64 ± 0.50	
Hip Add./Abd.	3.45 ± 0.52	2.45 ± 0.52	
Knee Fle./Ext.	3.09 ± 0.30	2.55 ± 0.52	
Ankle D.Fle./P.Fle.	2.72 ± 0.47	1.82 ± 0.60	

*Note:* *p* values represent tests for homogeneity. CVA type: cerebrovascular accident.

Abbreviations: Abd./Add, abductor/adductor; BBS, Berg Balance Scale; D.Fle./P.Fle., drosi/plantar flexor; Fle./Ext., flexor/extensor; HBG, high‐balance group; Hemo., hemorrhage; Inf., infarction; Injury dur. (mo.), injury duration (months); LBG, low‐balance group; MAS, Modified Ashworth Scale; mFAC, modified functional ambulatory category (1: layer; ~7: outdoor walker); MMT, manual muscle testing (5: normal; ~0: zero); TUG, timed up and go test; W/C, wheelchair.

^a^Values: mean ± standard deviation.

### 2.2. Experimental Conditions and E‐KAFO Device Description

#### 2.2.1. BP_C

In the BP_C, participants used a conventional AFO to prevent foot dragging, which commonly occurs due to impaired ankle dorsiflexion in patients with an ankle manual muscle testing (MMT) score below 3. The AFO stabilized the ankle joint and reduced the risk of foot drop but provided no active assistance to the knee or hip joints; thus, all lower limb movement relied on the participant’s own muscular effort. Consequently, changes observed in BP_C across the seven training sessions reflected *rehabilitative improvements* rather than immediate assistive effects.

#### 2.2.2. E‐KAFO_C

In the E‐KAFO_C, participants walked using an E‐KAFO, a rehabilitation‐assistive device designed to enhance gait stability and efficiency in individuals with poststroke hemiplegia. Based on the structural framework of a conventional KAFO, the E‐KAFO incorporates an electrically powered actuation system that detects walking intention through inertial and joint angle sensors. It then provides assistive torque to the knee joint via an electric motor, enabling controlled knee flexion during swing and stabilization during stance.

The device compensates for lower limb muscle weakness not addressed by passive AFOs and can be height‐adjusted without custom fabrication. The E‐KAFO used in this study was a precommercial prototype developed for research and development purposes by the Rehabilitation Engineering Research Institute, Korea Workers’ Compensation & Welfare Service. The E‐KAFO was used exclusively for research purposes in this study and was not a commercially marketed product at the time of investigation.

Participants underwent a brief familiarization session before the E‐KAFO_C assessment to reduce immediate adaptation bias. As these evaluations were short term, the outcomes primarily represent assistive rather than rehabilitative effects.

### 2.3. Device Characteristics

The total device weight was approximately 3.8 kg, comprising a knee actuator (1.1 kg), adjustable leg and ankle modules (2.2 kg), a battery pack (0.4 kg), and a waist strap (0.1 kg). The actuator module, responsible for assisting and controlling knee motion, contained a gear reducer, motor, sensors, and communication units, operated by an STM32F405 microcontroller and a Maxon EC motor driver. It was securely mounted to the thigh support frame, with its mechanical axis aligned precisely with the anatomical knee joint. A calibration procedure (offset correction in a static posture) ensured accurate alignment before use. The actuator included an IMU on the thigh‐aligned module and an absolute encoder at the knee joint axis to enable precise motion detection.

Based on sensor inputs, the system estimated user intent and generated a negative damping torque proportional to knee angular velocity, producing adaptive and flexible assistance. The E‐KAFO represents a knee‐actuated microprocessor‐controlled KAFO (MPCKAFO)—a subtype within the established MPCKAFO taxonomy—comparable to commercial systems such as the Ottobock C‐Brace and Agilik. A five‐state finite state machine (FSM) governed gait control, with transitions defined by IMU‐derived roll and pitch angles and knee angular velocity thresholds. This design was adapted from the FSM framework proposed by Heo et al. [21], thereby situating our approach within an established orthotic control paradigm and enhancing reproducibility.

During the swing phase, the E‐KAFO assisted natural knee flexion to promote physiological gait patterns. During stance, a spring–damper model stabilized the knee and prevented sudden buckling, reducing fall risk. Gait phase detection and walking intent were determined from hip and knee joint angle data on the affected side. The negative damping coefficient was individually tuned during familiarization by a physical therapist to ensure safety and comfort. Additionally, assistive force was automatically modulated in proportion to walking speed.

The knee joint range of motion was 0°–120°, and the ankle joint range was −10° to 10°. The actuator generated up to 14 Nm of assistive torque at the knee joint. Power was supplied by a rechargeable 24 VDC, 3500 mAh lithium battery (AC 100–240 V input and 25.2 VDC output).

The battery pack was positioned near the body’s center of mass, worn at the waist to minimize perceived load and improve comfort. The motor and reducer were arranged in parallel and connected through a pulley–belt system to reduce device thickness and shift the center of mass closer to the body.

An embedded overcurrent sensor automatically shut off power in the event of excessive current or low voltage, ensuring safety. The neoprene waist strap and thigh section effectively distributed device weight, minimized abnormal hip motion, and promoted a more natural gait.

**Figure 1:**
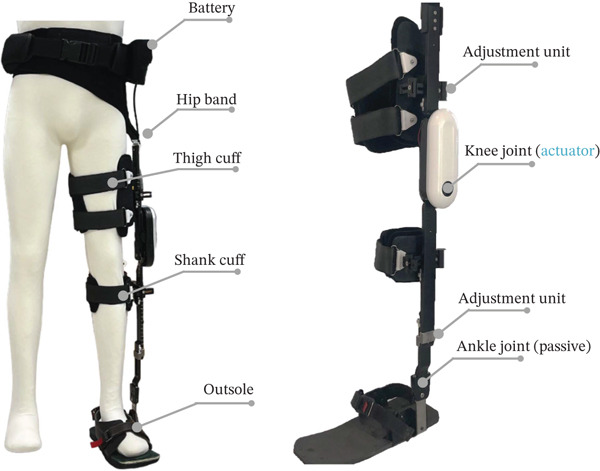
E‐KAFO system.

Although structurally similar to other KAFO‐based powered orthoses, this device’s novelty lies in its clinical application and functional validation in individuals with hemiplegia. Its explicit state transitions, sensor calibration, individualized tuning, and precise control within the MPCKAFO framework allow phase‐specific, adaptive assistance that enhances both gait performance and safety. Device components and control logic are summarized in Table 2 and illustrated in Figure 1.

**Table 2:** Comprehensive technical specifications of the E‐KAFO system.

Component	Specification
Total weight	3.8 kg
Component weights	Knee actuator: 1.13 kg Adjustable parts and ankle: 2.2 kg Battery pack: 0.4 kg Waist strap: 0.1 kg
Dimensions	(W)120 mm × (D)320 mm × (H)760 ~ 890 mm
Range of motion	Knee joint: 0°–120° Ankle joint: −10° to 10°
Maximum assistance force	14 Nm (knee joint)
Battery specifications	29.5 V/3.5 Ah (24 VDC, 3500 mAh) Charging: AC–DC adapter Input: AC 100–240 VAC Output: 25.2 VDC
Control system	STM32F405 microcontroller Maxon EC motor driver

### 2.4. Intervention Protocol

To assess the short‐term effects of the E‐KAFO, participants completed a 3‐week intervention program comprising seven training sessions conducted two to three times per week. Each session consisted of approximately 10 min for device donning and adaptation, 40 min of gait‐focused robotic training, and a 10‐min cooldown period with device removal. A safety harness system was employed throughout the sessions to prevent falls.

Gait function and physiological performance were evaluated using the 5‐m walk test (5‐mWT), 3‐min walk test (3‐MWT), and 6‐min walk test (6‐MWT), administered at pre‐ and postintervention time points. Outcomes obtained under the E‐KAFO_C primarily reflected short‐term assistive effects following initial familiarization, whereas those under the body‐powered AFO condition (BP_C) represented rehabilitative improvements independent of device assistance.

**Figure 2:**
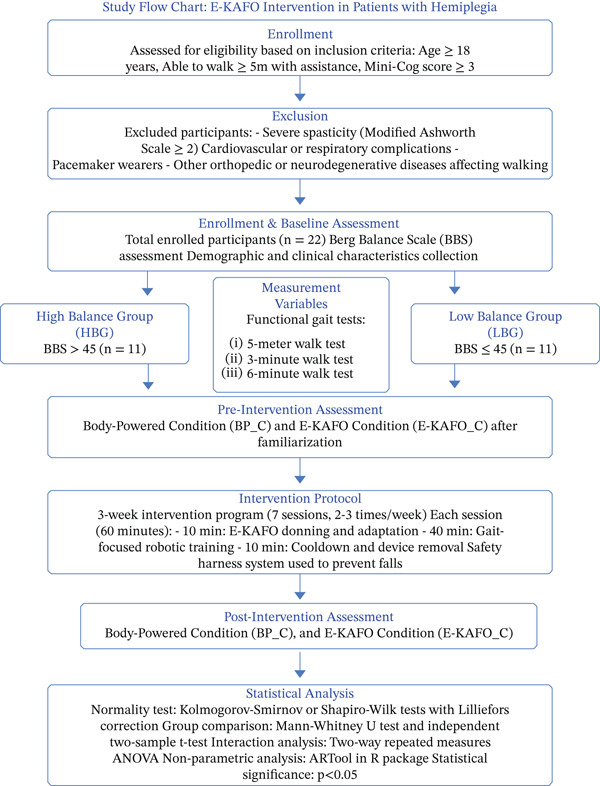
Flow chart.

By clearly distinguishing between E‐KAFO_C (assistive efficacy) and BP_C (rehabilitative *effectiveness*), this study enabled a precise interpretation of the E‐KAFO’s immediate functional support relative to the progressive gains achieved through repeated gait training sessions. The preintervention assessment under the E‐KAFO_C was conducted after ensuring proper alignment between the device actuator and the participant’s anatomical knee joint axis, as well as adequate adaptation to the device. Postintervention assessments were performed upon completion of the full 3‐week training program (Figure 2).

Concomitant physiotherapy was implemented based on routine clinical practice rather than a formal study protocol. Specifically, all participants continued only their pre‐existing low‐intensity physiotherapy as part of standard care during the study period, and no powered orthoses or new gait or balance training programs were initiated specifically for this study. Therapists were instructed to avoid substantive modifications to ongoing regimens. Furthermore, the frequency, structure, and duration of these concomitant sessions were not standardized or prospectively recorded. The laboratory environment was maintained at a temperature of 22°C–24°C and a relative humidity of 40%–60%, in accordance with the recommendations of Wolkoff et al.; this was done to minimize environmental variability and ensure optimal conditions for gait evaluation [31].

### 2.5. Progression Criteria and Parameterization

Following established stroke rehabilitation guidelines [7, 32–34], training was processed as per predefined clinical criteria rather than arbitrary therapist judgement. During each 40‐min E‐KAFO session, therapists adjusted walking speed, walking distance/duration, and device assistance according to standardized stepwise rules while continuously monitoring gait quality and physiological load. At each session, the therapist first confirmed that the participant could safely complete the current walking duration and distance without undue fatigue, pain, near‐falls, or marked deterioration in gait quality (e.g., loss of foot clearance, knee buckling or excessive hyperextension, compensatory trunk sway, or other abnormal patterns).

When these conditions were met in at least two consecutive sessions, walking distance or training duration was increased in small, stepwise increments (approximately 5%–10% per progression), and manual or handrail assistance was gradually reduced. If signs of instability, overexertion, or gait deterioration emerged, the training parameters were maintained or reduced to a previous tolerable level. All parameter adjustments, deviations from the planned progression, and any adverse events were documented to support protocol transparency and reproducibility.

### 2.6. Safety and Usability Monitoring

In this pilot study, safety and usability were monitored pragmatically during each session. For example, therapists documented notable issues (e.g., near‐falls, skin irritation/pressure, excessive fatigue, donning/doffing difficulty, and device alarms) in routine session notes. No standardized adverse event classification system or validated usability scales were administered.

Safety and usability were monitored prospectively at each session using a standardized case report form. The form captured participant and session identifiers (ID, date, and training condition), event classification (device malfunction, usability discomfort, and adverse event), timing and duration, and a concise narrative description (e.g., controller alarm during midstance, and strap slippage after 5 min). Events were graded for severity (mild/moderate/severe based on functional impact and need for intervention) and adjudicated for relatedness (device‐related, nondevice, and indeterminate); relevant physiological indicators (e.g., heart rate and RPE), actions taken (parameter adjustment, rest, fit modification, and session termination), and outcomes (resolved, continued observation, and referral), including recurrence, were recorded. For malfunctions, device metadata (device ID/firmware and battery status) and reproducibility were documented. Assessor–therapist separation was maintained throughout the study.

All training sessions were delivered by treating therapists, while outcome assessments were conducted by independent assessors who had no involvement in intervention delivery. Assessors were blinded to participants’ BBS‐based subgroup assignment and to the study hypotheses. Complete blinding to device condition (E‐KAFO vs. body‐powered) was not feasible due to its visible nature; to mitigate potential bias, assessors followed standardized scripts and timing procedures, and objective measures (time, distance, and heart rate) were used.

### 2.7. BBS

Balance in participants with hemiplegia was assessed using the BBS. In this test, the evaluator rates the participants’ ability to maintain balance while performing 14 functional tasks related to daily living using a 5‐point ordinal scale. The BBS is known for its high validity and reliability and moderate responsiveness. The scores range from 0 to 56, with a score of 56 indicating functional balance. A score of ≤ 45 suggests a higher risk of falling [29, 30, 35]. Participants were divided into two groups based on a cutoff score of 45, and the intervention’s short‐term effect on the E‐KAFO was investigated accordingly.

### 2.8. Assessment of Gait Function

The 5‐mWT was selected to measure the walking speed [36–48].

This test has demonstrated high reliability in patients with stroke. A 10‐m path was marked on the floor with designated segments: acceleration (0–2.5 m), measurement (2.5–7.5 m), and deceleration (7.5–10 m) zones. Visual markers were placed at each point to facilitate recognition. After the participants became familiar with the course at a comfortable pace, the test was conducted at maximum walking speed. A digital stopwatch was used to measure the time from the moment the participants crossed the start line of the measurement zone (2.5 m) to the end line (7.5 m).

To precisely evaluate the walking mechanics of patients with hemiplegia, the 3‐MWT and 6‐MWT were conducted sequentially. The 3‐MWT [39–41] and 6‐MWT [42, 43] are gait endurance tests that measure the distance walked within a given time. The 3‐MWT evaluates short‐term walking performance and initial adaptation ability. For patients with low walking endurance or high cardiovascular burden, performing the 6‐MWT may be challenging. Therefore, to ensure participant safety, the 3‐MWT was conducted first, followed by the 6‐MWT after confirming walking adaptability, allowing for a more reliable assessment.

Both tests were performed on an indoor flat corridor floor. A 20‐m path was marked on the floor with tape, and cones were placed at the start and end points to guide the participants along the designated route. The participants walked continuously at a self‐selected pace for 3 and 6 min. Before the test, each participant underwent a practice trial to ensure that they understood the instructions. Time was recorded using a stopwatch, and the distance walked was measured at 3 and 6 min. During the test, a clinical professional followed each participant to ensure safety, while offering verbal encouragement. Prior to participation, the participants were allowed sufficient rest and were rebriefed that the trial would last for 3 and 6 min, respectively.

The following variables were recorded at each time point: At baseline, participants’ resting heart rate was checked before starting. At 3‐min point, the distance walked without interruption was recorded, and the participant’s condition was reassessed using heart rate. At 6‐min point, the total walking distance was recorded, and the participant’s condition was again assessed using heart rate.

### 2.9. PCI

Each participant performed the 3‐MWT and 6‐MWT at a comfortable walking speed along an experimental walkway. To measure heart rate during walking, POLAR SENSE (Polar Electro Inc., United States) sensors were attached near the upper arm. The researcher measured each participant’s walking and resting heart rates (beats/min), and walking speed was calculated by considering both walking time and distance covered. To measure the resting heart rate, the participants maintained a stable position, lying down comfortably for 10 min. Walking heart rate was measured twice during the 3‐MWT and 6‐MWT under both BP_C and E‐KAFO_C. Additionally, to assess walking energy efficiency, the PCI was calculated using the following formula (Equation 1).

(1) PCI=walking heart rate−resting heart rate beats/minwalking speed meter/min

Equation1shows the physiological cost index (beats/meter).

The PCI is an indicator that reflects the increase in heart rate per unit distance (meter) and is used to indirectly assess energy expenditure during walking. A lower PCI value indicates higher walking efficiency and plays an important role in quantitatively comparing the physical burden associated with the use of walking‐assistive devices [44, 45].

### 2.10. Statistical Analysis

The normality of the data was assessed using the Kolmogorov–Smirnov or Shapiro–Wilk tests, with Lilliefors correction applied. All data were complete prior to analysis, with no missing values observed (*n* = 22). Group homogeneity was assessed using the Mann–Whitney *U* test and independent two‐sample *t*‐test, as appropriate. Two‐way repeated measures analysis of variance (ANOVA) was performed to investigate time × group interactions. For nonparametric two‐way ANOVA, the ARTool in R was applied to convert results to ranked data. Statistical significance was set at *p* < 0.05. Experimental data are presented as means, standard deviations, and %Diff (post–pre difference/pretest × 100). Data entry, processing, and analyses were conducted using SPSS 18.0 (IBM, Armonk, New York, United States), R, and Microsoft Excel 2013 (Microsoft, United States).

These procedures confirmed data normality, evaluated group homogeneity, assessed the utility of the assistive device, and ensured robust outcome analysis. Effect sizes were calculated to quantify the magnitude of within‐group pre–post changes. Specifically, Cohen’s *d* was computed for the main gait and PCI outcomes, with effect sizes of 0.2, 0.5, and 0.8 interpreted as small, medium, and large, respectively.

For clinical interpretability, pre–post changes were compared against published minimal detectable change (MDC) and minimal clinically important difference (MCID) thresholds for stroke populations. Gait functional outcomes were evaluated relative to stroke‐specific MDC/MCID values, and gait speed changes referenced 10‐mWT benchmarks as contextual comparators for our 5‐mWT. The proportion of participants exceeding MDC/MCID thresholds is reported, with sources and applicability detailed in table footnotes.

## 3. Results

### 3.1. Time of 5‐mWT at Maximal Pace (Second)

In the BP_C condition, walking time decreased by 15.16% in the HBG (from 5.83 ± 1.68 to 4.93 ± 1.37 s, *p* = 0.104, Cohen’s *d* = −0.574, 95% CI [−1.360, −0.400]), whereas the LBG exhibited a significant 18.91% reduction (from 10.04 ± 3.67 to 8.15 ± 2.55 s, *p* = 0.002, Cohen’s *d* = −0.602, 95% CI [−3.489, −0.309]). Both groups demonstrated medium to large effect sizes for time reduction. At baseline, the LBG had significantly longer walking times than the HBG (*p*_group = 0.004), and this difference remained postintervention (*p*_group = 0.002). The interaction effect between time and group (*T*∗*G*, *F* = 1.759, *p* = 0.200) was not significant, indicating that both groups responded similarly to the body‐powered intervention regardless of initial balance ability (Table 3).

**Table 3:** Results of body‐powered condition through two‐way repeated measures ANOVA (*n* = 22).

	Group	Pre	Post	%Diff	Cohen’s *d*	*p*_time	So.	*F*	*p*	*Δ*	Exceeds MDC	Exceeds MCID
5‐mWT time (s)	HBG (*n* = 11)	5.83 ± 1.68^a^	4.93 ± 1.37	−15.16	−0.574	0.104	*T*	13.957	0.001	−0.90	^g^	^g^
	LBG (*n* = 11)	10.04 ± 3.67	8.15 ± 2.55	−**18.91** ^∗^	−0.602	0.002	*G*	14.243	0.001	−1.89	^g^	^g^
	*p* *_Group*	0.004	0.002				*T*∗*G*	1.759	0.200			
5‐mWT speed (m/s)	HBG (*n* = 11)	0.95 ± 0.37	1.10 ± 0.36	**+14.9** ^∗^	0.401	< 0.001	*T*	31.000	< 0.001	+0.15	No^c^	No^b^
	LBG (*n* = 11)	0.56 ± 0.18	0.66 ± 0.19	**+19.6** ^∗^	0.592	0.004	*G*	12.393	0.002	+0.10	No^c^	No^b^
	*p* *_Group*	0.006	0.002				*T*∗*G*	0.806	0.380			
3‐MWT distance (m)	HBG (*n* = 11)	144.47 ± 36.58	155.71 ± 38.53	+7.78	0.299	0.087	*T*	9.933	0.005	+11.24	^f^	^f^
	LBG (*n* = 11)	90.74 ± 32.24	107.35 ± 34.42	**+18.31** ^∗^	0.498	0.015	*G*	12.416	0.002	+16.61	^f^	^f^
	*p* *_Group*	0.002	0.006				*T*∗*G*	0.371	0.549			
3‐MWT PCI (beats/m)	HBG (*n* = 11)	0.50 ± 0.19	0.42 ± 0.14	−15.62	−0.468	0.194	*T*	9.219	0.007	−0.08	^f^	^f^
	LBG (*n* = 11)	0.79 ± 0.33	0.61 ± 0.19	−**22.33** ^∗^	−0.655	0.008	*G*	7.677	0.012	−0.18	^f^	^f^
	*p* *_Group*	0.024	0.016				*T*∗*G*	1.287	0.270			
6‐MWT distance (m)	HBG (*n* = 11)	289.19 ± 74.61	309.64 ± 79.14	+7.07	0.266	0.115	*T*	9.644	0.006	+20.45	No^d^	No^b^
	LBG (*n* = 11)	177.09 ± 65.37	211.11 ± 66.31	+19.21 ^∗^	0.517	0.013	*G*	12.973	0.002	+34.02	No^d^	Yes^b,e^
	*p* *_Group*	0.001	0.005				*T*∗*G*	0.599	0.448			
6‐MWT PCI (beats/m)	HBG (*n* = 11)	0.50 ± 0.19	0.42 ± 0.14	−15.12	−0.462	0.170	*T*	11.764	0.003	−0.08	^f^	^f^
	LBG (*n* = 11)	0.80 ± 0.30	0.62 ± 0.19	−**22.35** ^∗^	−0.714	0.003	*G*	8.855	0.007	−0.18	^f^	^f^
	*p* *_Group*	0.012	0.013				*T*∗*G*	2.007	0.172			

*Note:* Significant results are highlighted in bold. *p* *_time*: *p* value between pre‐ and postresult; *p* *_Group*: *p* value between LBG and HBG results at pre‐ and posttime points; < 0.001, *p* value less than 0.001. PCI, physiological cost index (mean HR at work [bpm/min] − mean HR at rest [bpm/min])/walking speed (m/min); %Diff, post–pre/preresult (%); *Δ*, absolute pre–post change (postvalue minus prevalue). Effect size: Cohen’s *d* is reported for within‐group pre–post comparisons (thresholds: 0.2 = small, 0.5 = medium, and 0.8 = large effect).

Abbreviations: 3‐MWT, 3‐min walk test at self‐selected gait speed; 5‐mWT: 5‐m walk test at maximal pace; 6‐MWT: 6‐min walk test at self‐selected gait speed; BBS, Berg Balance Scale; E‐KAFO, wearing electric knee–ankle–foot orthosis; HBG, high‐balance group; LBG, low‐balance group; PCI, physiological cost index; So., source about interaction effect; *T*∗*G*, time∗group.

^a^Values: mean ± standard deviation.

^b^Values: MCID (minimal clinically important difference, gait speed): 10‐mWT anchor‐based benchmark ≈ 0.16 m/s, used contextually for 5‐mWT speed [46].

^c^Values: MDC (minimal detectable change, gait speed) varies by baseline speed; typical 10‐mWT strata: > 0.8 m/s ≈ 0.21 m/s and 0.4–0.8 m/s ≈ 0.11 m/s [47].

^d^Values: 6‐MWT MDC: stroke co[49]hort MDC90 ≈ 54.1 m [50].

^e^Values: *Δ* is near a commonly cited lower MCID bound (~44 m) for slower walkers; interpret cautiously [49].

^f^Values: 3‐MWT/PCI: no widely established stroke‐specific MDC/MCID for 3‐MWT; PCI MDC95~0.52 beats/m has been reported but remains heterogeneous—reported here descriptively [45, 51].

^g^Values: not established/not computed.

^∗^ *p* < 0.05.

In the E‐KAFO_C condition, walking time decreased postintervention in both groups. The LBG showed a significant reduction of 5.65% (*p* < 0.001, Cohen’s *d* = −0.884, 95% CI [−5.618, −1.218]), while the HBG demonstrated a nonsignificant reduction of 6.87% (*p* = 0.276, Cohen’s *d* = −0.465, 95% CI [−1.152, −0.433]). Notably, the LBG exhibited a large effect size, compared with a medium effect size in the HBG. Group comparisons of pre‐ and postintervention differences (*p*_group) were not significant at either time point (pre: *p* = 0.277; post: *p* = 0.251).

Importantly, the interaction effect (*T*∗*G*, *F* = 6.834, *p* = 0.017) was significant in contrast to the nonsignificant interaction observed in BP_C. This indicates that initial balance ability differentially influenced the effectiveness of the E‐KAFO intervention, with the LBG showing greater responsiveness, although both groups achieved clinically meaningful improvements (Table 4).

**Table 4:** Results of E‐KAFO condition through two‐way repeated measures ANOVA (*n* = 22).

	Group	Pre	Post	%Diff	Cohen’s *d*	*p*_time	So.	*F*	*p*	*Δ*	Exceeds MDC	Exceeds MCID
5‐mWT time (s)	HBG (*n* = 11)	7.24 ± 1.32^a^	6.74 ± 1.85	−6.87	−0.465	0.276	*T*	17.622	< 0.001	−0.50	^g^	^g^
	LBG (*n* = 11)	8.64 ± 2.36	8.15 ± 0.71	−**5.65**∗	−0.884	< 0.001	*G*	11.954	0.002	−0.49	^g^	^g^
	*p* *_Group*	0.277	0.251				*T*∗*G*	6.834	**0.017**			
5‐mWT speed (m/s)	HBG (*n* = 11)	0.85 ± 0.17	0.98 ± 0.32	**+15.28**∗	0.442	< 0.001	*T*	61.250	< 0.001	+0.13 m/s	No^c^	No^b^
	LBG (*n* = 11)	0.49 ± 0.17	0.65 ± 0.17	**+33.31**∗	0.937	< 0.001	*G*	10.540	0.004	+0.16 m/s	Yes^c^	Yes^b^
	*p* *_Group*	0.002	0.008				*T*∗*G*	1.250	0.277			
3‐MWT distance (m)	HBG (*n* = 11)	125.37 ± 25.15	134.16 ± 29.69	**+7.01** ∗	0.319	0.046	*T*	26.515	< 0.001	+8.79 m	^f^	^f^
	LBG (*n* = 11)	83.66 ± 36.09	105.01 ± 34.30	**+25.51**∗	0.606	< 0.001	*G*	7.260	0.014	+21.35 m	^f^	^f^
	*p* *_Group*	0.006	0.046				*T*∗*G*	4.602	0.044			
3‐MWT PCI (beats/m)	HBG (*n* = 11)	0.59 ± 0.18	0.57 ± 0.21	−2.95	−0.088	0.841	*T*	4.040	0.058	−0.02	^f^	^f^
	LBG (*n* = 11)	0.92 ± 0.55	0.69 ± 0.30	−**25.40**∗	−0.529	0.016	*G*	2.915	0.103	−0.23	^f^	^f^
	*p* *_Group*	0.081	0.302				*T*∗*G*	2.965	0.101			
6‐MWT distance (m)	HBG (*n* = 11)	247.49 ± 54.78	291.46 ± 50.12	**+17.77** ∗	0.308	0.047	*T*	27.856	< 0.001	+43.97 m	No^d^	^e,b^
	LBG (*n* = 11)	167.71 ± 74.72	203.90 ± 19.43	**+21.58**∗	0.636	< 0.001	*G*	7.414	0.013	+36.19 m	No^d^	Yes^b^
	*p* *_Group*	0.013	0.003				*T*∗*G*	5.237	**0.033**			
6‐MWT PCI (beats/m)	HBG (*n* = 11)	0.58 ± 0.18	0.57 ± 0.21	−2.79	−0.084	0.875	*T*	3.335	0.083	−0.01	^f^	^f^
	LBG (*n* = 11)	0.79 ± 0.28	0.64 ± 0.27	−**19.13**∗	−0.587	0.025	*G*	3.013	0.098	−0.15	^f^	^f^
	*p* *_Group*	0.087	0.334				*T*∗*G*	2.560	0.125			

*Note:* Significant results are highlighted in bold. *p*_time: *p* value between pre‐ and postresult; *p*_Group: *p* value between LBG and HBG results at pre‐ and posttime points; < 0.001: *p* value less than 0.001; PCI, physiological cost index (mean HR at work [bpm/min] − mean HR at rest [bpm/min])/walking speed (m/min); %Diff, post–pre/preresult (%); *Δ*, absolute pre–post change (postvalue minus prevalue). Effect size: Cohen’s *d* is reported for within‐group pre–post comparisons (thresholds: 0.2 = small, 0.5 = medium, and 0.8 = large effect).

Abbreviations: 3‐MWT: 3‐min walk test at self‐selected gait speed; 5‐mWT: 5‐m walk test at maximal pace; 6‐MWT: 6‐min walk test at self‐selected gait speed; BBS, Berg Balance Scale; E‐KAFO, wearing electric knee–ankle–foot orthosis; HBG, high‐balance group; LBG, low‐balance group; PCI, physiological cost index; So., source about interaction effect; *T*∗*G*, time∗group.

^a^Values: mean ± standard deviation.

^b^Values: MCID (minimal clinically important difference, gait speed): 10‐mWT anchor‐based benchmark ≈ 0.16 m/s, used contextually for 5‐mWT speed [46].

^c^Values: MDC (minimal detectable change, gait speed) varies by baseline speed; typical 10‐mWT strata: > 0.8 m/s ≈ 0.21 m/s and 0.4–0.8 m/s ≈ 0.11 m/s [47].

^d^Values: 6‐MWT MDC: stroke co[49]hort MDC90 ≈ 54.1 m [50].

^e^Values: *Δ* is near a commonly cited lower MCID bound (~44 m) for slower walkers; interpret cautiously [49].

^f^Values: 3‐MWT/PCI: no widely established stroke‐specific MDC/MCID for 3‐MWT; PCI MDC95~0.52 beats/m has been reported but remains heterogeneous—reported here descriptively [45, 51].

^g^Values: not established/not computed.

∗ *p* < 0.05.

### 3.2. Speed of 5‐mWT at Maximal Pace (Meter per Second)

In the BP_C condition, walking speed increased significantly by 14.9% in the HBG (from 0.95 ± 0.37 to 1.10 ± 0.36 m/s, *p* < 0.001, Cohen’s *d* = 0.401, 95% CI [0.052, 0.232]) and by 19.6% in the LBG (from 0.56 ± 0.18 to 0.66 ± 0.19 m/s, *p* = 0.004, Cohen’s *d* = 0.592, 95% CI [0.035, 0.183]). Both groups achieved medium effect sizes, with the LBG showing a larger effect. Walking speed was significantly different between HBG and LBG at both preintervention (*p* = 0.006) and postintervention (*p* = 0.002) time points, indicating that the HBG maintained higher speeds throughout. The interaction effect (*T*∗*G*, *F* = 0.806, *p* = 0.380) was not significant, confirming comparable responsiveness across groups (Table 3 and Figure 3a).

Figure 3(a, b) 5‐mWT speed both BP_C and E‐KAFO_C.

**Figure:**
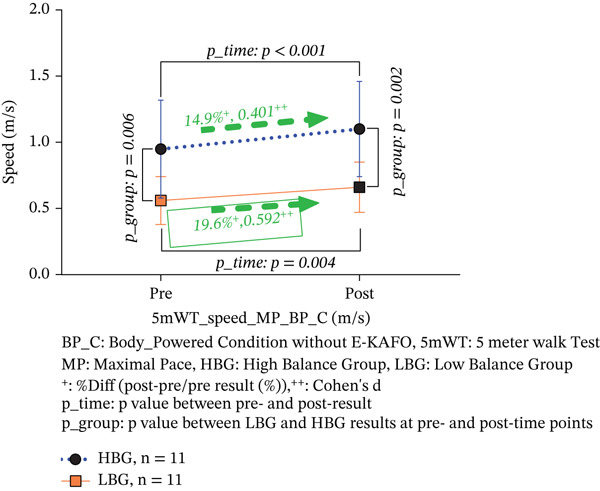
(a) 5‐mWT speed about BP_C

**Figure:**
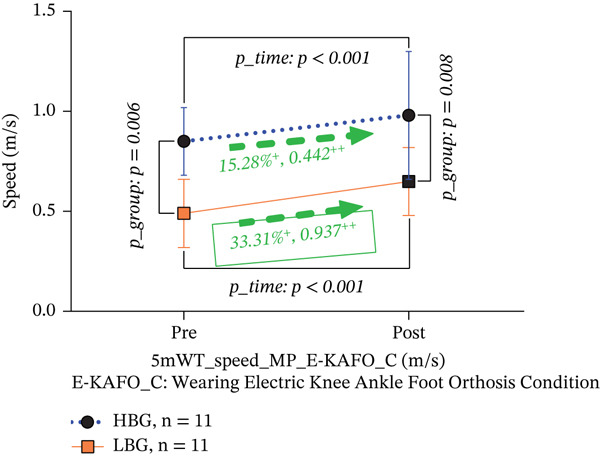
(b) 5‐mWT speed about E‐KAFO_C

In the E‐KAFO_C, walking speed increased significantly by 15.28% in the HBG (from 0.85 ± 0.17 to 0.98 ± 0.32 m/s, *p* < 0.001, Cohen’s *d* = 0.442, 95% CI [0.079, 0.181]) and by 33.31% in the LBG (from 0.49 ± 0.17 to 0.65 ± 0.17 m/s, *p* < 0.001, Cohen’s *d* = 0.937, 95% CI [0.089, 0.237]). The LBG demonstrated a large effect size compared with the medium effect size in the HBG, suggesting greater responsiveness in participants with lower initial balance. Significant differences between groups persisted at preintervention (*p* = 0.002) and postintervention (*p* = 0.008). The interaction effect was not statistically significant (*T*∗*G*, *F* = 1.250, *p* = 0.277), indicating that despite differences in effect sizes, the formal interaction did not reach significance (Table 4 and Figure 3b).

### 3.3. Distance of 6‐MWT at Self‐Selected Pace (Meter)

In the BP_C, the HBG showed a 7.07% increase in distance (from 289.19 ± 74.61 to 309.64 ± 79.14 m), which was not statistically significant (*p* = 0.115, Cohen’s *d* = 0.266, 95% CI [−12.368, 53.259]). The LBG demonstrated a significant 19.21% improvement (from 177.09 ± 65.37 to 211.11 ± 66.31 m, *p* = 0.013, Cohen’s *d* = 0.517, 95% CI [12.800, 55.236]). The HBG effect size was small, while the LBG achieved a medium effect size. Between‐group differences remained significant at both preintervention (*p* = 0.001) and postintervention (*p* = 0.005). The interaction effect was not significant (*T*∗*G*, *F* = 0.599, *p* = 0.448), indicating parallel improvement patterns despite the LBG’s greater absolute gains (Table 3 and Figure 4a).

Figure 4(a, b) 6‐MWT distance both BP_C and E‐KAFO_C.

**Figure:**
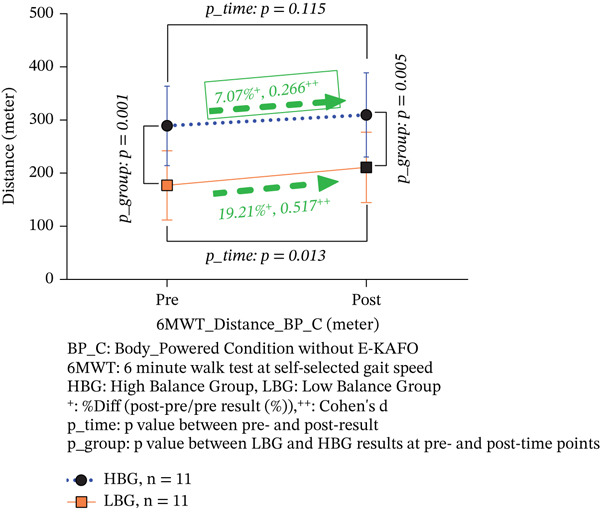
(a) 6‐MWT distance about BP_C

**Figure:**
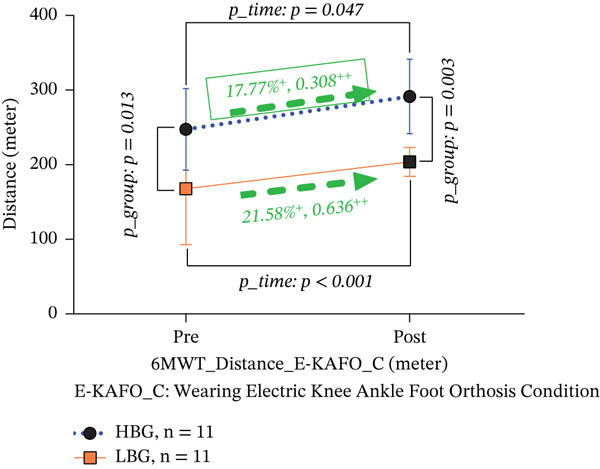
(b) 6‐MWT distance about E‐KAFO_C

In the E‐KAFO_C, the HBG showed a significant 17.77% increase (from 247.49 ± 54.78 to 291.46 ± 50.12 m, *p* = 0.047, Cohen’s *d* = 0.308, 95% CI [−0.962, 36.199]) and the LBG a 21.58% increase (from 167.71 ± 74.72 to 203.90 ± 19.43 m, *p* < 0.001, Cohen’s *d* = 0.636, 95% CI [26.031, 63.151]). The HBG showed a small‐to‐medium effect, and the LBG showed a medium‐to‐large effect size. Pre‐ and postintervention differences between groups remained significant (pre: *p* = 0.013; post: *p* = 0.003). Importantly, the interaction effect was significant (*T*∗*G*, *F* = 5.237, *p* = 0.033), indicating that the E‐KAFO intervention produced greater improvements in participants with lower baseline balance (Table 4 and Figure 4b).

### 3.4. PCI of 6‐MWT at Self‐Selected Pace (Beats/Meter)

In the BP_C, the HBG showed a 15.12% reduction in PCI (from 0.50 ± 0.19 to 0.42 ± 0.14 beats/m), which was not statistically significant (*p* = 0.170, Cohen’s *d* = −0.462, 95% CI [−0.161, 0.009]). The LBG demonstrated a significant 22.35% reduction (from 0.80 ± 0.30 to 0.62 ± 0.19 beats/m, *p* = 0.003, Cohen’s *d* = −0.714, 95% CI [−0.317, −0.039]). The HBG effect size was medium with CIs crossing zero, whereas the LBG achieved a large effect with CIs excluding zero. Between‐group differences were significant at pre‐ (*p* = 0.012) and postintervention (*p* = 0.013). The interaction effect was not significant (*T*∗*G*, *F* = 2.007, *p* = 0.172), indicating consistent improvement trends across groups (Table 3 and Figure 5a).

Figure 5(a, b) 6‐MWT PCI both BP_C and E‐KAFO_C.

**Figure:**
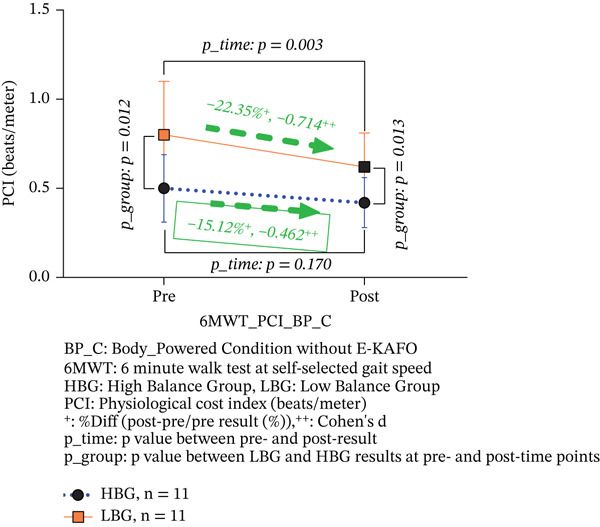
(a) 6‐MWT PCI about BP_C

**Figure:**
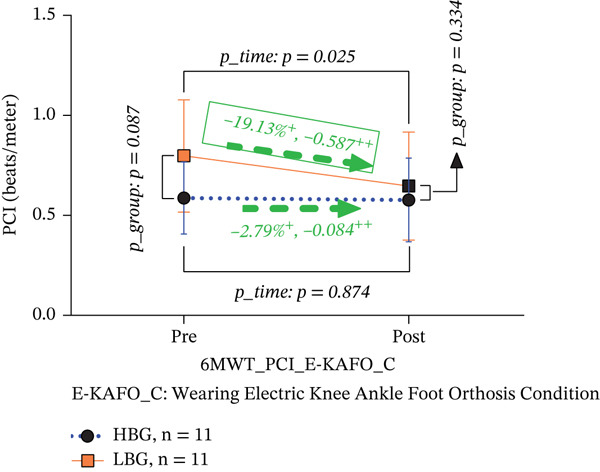
(b) 6‐MWT PCI about E‐KAFO_C

In the E‐KAFO_C, the HBG showed a negligible 2.79% reduction in PCI (from 0.58 ± 0.18 to 0.57 ± 0.21 beats/m, *p* = 0.875, Cohen’s *d* = −0.084, 95% CI [−0.159, 0.126]), whereas the LBG exhibited a significant 19.13% reduction (from 0.79 ± 0.28 to 0.64 ± 0.27 beats/m, *p* = 0.025, Cohen’s *d* = −0.587, 95% CI [−0.603, 0.052]). The HBG effect was negligible, and the LBG showed a medium effect, indicating differential responsiveness. Between‐group differences were not significant pre‐ (*p* = 0.087) or postintervention (*p* = 0.334). The interaction effect was not statistically significant (*T*∗*G*, *F* = 2.560, *p* = 0.125), although the moderate *F*‐value suggests a trend toward differential effects (Table 4 and Figure 5b).

### 3.5. Comparison Between BP_C and E‐KAFO_C

As summarized in Table 5, both the BP_C and the E‐KAFO_C yielded short‐term improvements in gait outcomes, with LBG driving more pronounced changes. In the HBG, both BP_C and E‐KAFO_C led to significant increases in 5‐mWT speed (*Δ* ≈ 0.13–0.15 m/s, small–medium effect sizes), but these changes did not exceed MDC or MCID. For 6‐MWT distance, only E‐KAFO_C produced a statistically significant gain (+43.97 m vs. +20.45 m under BP_C), approaching but not reaching commonly cited MDC values.

**Table 5:** Comparison of pre–post changes between BP_C and E‐KAFO_C.

Outcome and group	Condition	*Δ*	Cohen’s *d*	*p*_time	Exceeds MDC	Exceeds MCID
5‐mWT speed (m/s), HBG	BP_C	+0.15	0.401	< 0.001	No^b^	No^a^
	E‐KAFO_C	+0.13	0.442	< 0.001	No^b^	No^a^
5‐mWT speed (m/s), LBG	BP_C	+0.10	0.592	0.004	No^b^	No^a^
	E‐KAFO_C	+0.16	0.937	< 0.001	Yes^b^	Yes^a^
3‐MWT distance (m), HBG	BP_C	+11.24	0.299	0.087	^e^	^e^
	E‐KAFO_C	+8.79	0.319	0.046	^e^	^e^
3‐MWT distance (m), LBG	BP_C	+16.61	0.498	0.015	^e^	^e^
	E‐KAFO_C	+21.35	0.606	< 0.001	^e^	^e^
3‐MWT PCI (beats/m), LBG	BP_C	−0.18	−0.655	0.008	^e^	^e^
	E‐KAFO_C	−0.23	−0.529	0.016	^e^	^e^
6‐MWT distance (m), HBG	BP_C	+20.45	0.266	0.115	No^c^	No^a^
	E‐KAFO_C	+43.97	0.308	0.047	No^c^	^d,a^
6‐MWT distance (m), LBG	BP_C	+34.02	0.517	0.013	No^c^	Yes^a,d^
	E‐KAFO_C	+36.19	0.636	< 0.001	No^c^	Yes^a^
6‐MWT PCI (beats/m), LBG	BP_C	−0.18	−0.714	0.003	^e^	^e^
	E‐KAFO_C	−0.15	−0.587	0.025	^e^	^e^

*Note:* *p* *_time*, *p* value between pre‐ and postresult. PCI, physiological cost index (mean HR at work [bpm/min] − mean HR at rest [bpm/min])/walking speed (m/min); %Diff, post–pre/preresult (%); *Δ*, absolute pre–post change (postvalue minus prevalue). Effect size: Cohen’s *d* is reported for within‐group pre–post comparisons (thresholds: 0.2 = small, 0.5 = medium, and 0.8 = large effect).

Abbreviations: E‐KAFO, wearing electric knee–ankle–foot orthosis; HBG, high‐balance group; LBG, low‐balance group; PCI, physiological cost index.

^a^Values: MCID (minimal clinically important difference, gait speed): 10‐mWT anchor‐based benchmark ≈ 0.16 m/s, used contextually for 5‐mWT speed [46].

^b^Values: MDC (minimal detectable change, gait speed) varies by baseline speed; typical 10‐mWT strata: > 0.8 m/s ≈ 0.21 m/s and 0.4–0.8 m/s ≈ 0.11 m/s [47].

^c^Values: 6‐MWT MDC: stroke co[49]hort MDC90 ≈ 54.1 m [50].

^d^Values: *Δ* is near a commonly cited lower MCID bound (~44 m) for slower walkers; interpret cautiously [49].

^e^Values: 3‐MWT/PCI: no widely established stroke‐specific MDC/MCID for 3‐MWT; PCI MDC95~0.52 beats/m has been reported but remains heterogeneous—reported here descriptively [45, 51].

In the LBG, both conditions yielded significant improvements in 5‐mWT speed, 3‐MWT distance, and 6‐MWT distance, with consistently larger effect sizes under E‐KAFO_C. Notably, only E‐KAFO_C exceeded both MDC and MCID for 5‐mWT speed (+0.16 m/s vs. +0.10 m/s under BP_C), while both conditions exceeded MCID for 6‐MWT distance. Reductions in PCI during 3‐MWT and 6‐MWT in the LBG were of similar magnitude under BP_C and E‐KAFO_C, with medium to medium–large effect sizes and no established MDC/MCID thresholds for formal clinical interpretation.

## 4. Discussion

This study analyzed the impact of the E‐KAFO (knee‐actuated MPCKAFO) on gait performance and the PCI in patients with stroke, comparing the short‐term effects of the intervention between two groups with differing baseline balance abilities as assessed by the BBS. The results demonstrated improvements in gait function following the intervention, with the most pronounced benefits observed in participants with lower baseline balance. While both conditions showed gains consistent with general gait training effects, the powered condition produced greater improvements in selected outcomes, suggesting an incremental benefit beyond that provided by a body‐powered orthosis.

Outcomes under E‐KAFO_C primarily reflect the device’s short‐term assistive effects, whereas BP_C captures rehabilitation gains independent of device support. To delineate assistive versus rehabilitative contributions, we present a side‐by‐side synthesis of within‐condition changes and the between‐condition difference in change (*Δ*E‐KAFO_C and *Δ*BP_C), interpreted against available MDC and MCID benchmarks.

Consistent with this approach, improvements in LBG were larger under E‐KAFO_C than BP_C for several outcomes, and, where applicable, these changes were contextualized using MDC/MCID thresholds (with 10‐mWT serving as a comparator for 5‐mWT speed) [46, 47]. This framework clarifies which gains likely reflect general training effects versus the added value of powered assistance. The comparison between BP_C and E‐KAFO_C (Table 5) facilitates an understanding of how much of the observed benefit can be attributed to general gait practice versus the added effect of powered assistance.

Under BP_C, particularly in the LBG, moderate pre–post effect sizes for 3‐MWT and 6‐MWT distance, with 6‐MWT distance exceeding the MCID, show that repeated gait training and continued use of a BP orthosis alone can yield clinically meaningful improvements in walking endurance and speed in chronic stroke patients; additionally, these findings are consistent with previous reports that conventional KAFO‐supported gait training can enhance walking capacity and efficiency in this population.

Against this rehabilitative background, E‐KAFO_C training appears to provide an incremental assistive benefit for selected outcomes. In the LBG, the increase in 5‐mWT speed under E‐KAFO_C (+0.16 m/s, large effect size, exceeding both MDC and MCID) was greater than the corresponding change under BP_C (+0.10 m/s, medium effect size, below MDC/MCID), suggesting a technology‐related enhancement of short‐term gait speed beyond general training effects. Similarly, medium‐to‐medium–large effect sizes for 3‐MWT and 6‐MWT distance under E‐KAFO_C, in conjunction with gains slightly greater than those seen under BP_C, support a potential added advantage of powered knee assistance for walking endurance. In contrast, the comparable magnitude of PCI reductions under BP_C and E‐KAFO_C in the LBG suggests that improvements in energy efficiency may be largely attributable to nonspecific training and adaptation rather than by the electric knee joint alone. Overall, these outcomes support a complementary interpretation in which body‐powered orthoses and repeated gait practice provide a substantial rehabilitative foundation, while E‐KAFO‐assisted training may confer additional gains in functional gait, particularly in individuals with a low baseline balance.

From a physiological standpoint, the observed improvements under the E‐KAFO_C are likely driven by an interplay of neuromuscular control, compensatory kinematics, and energy expenditure. Conventional KAFOs have been shown to enhance gait ability in stroke patients by preventing knee buckling during stance and facilitating more appropriate activation of the quadriceps and other antigravity musculature, thereby supporting more stable weight acceptance on the paretic limb [48]. By providing active control of the knee joint throughout the gait cycle, the E‐KAFO may further reduce the need for maladaptive strategies, which are commonly manifested as a hemiparetic gait; these changes have been linked to an increase in mechanical work and thus energy expenditure [52]. Additionally, studies of powered ankle and stance‐control orthoses in neurological populations suggest that appropriately timed joint assistance can improve spatiotemporal symmetry and paretic propulsion, even when reductions in metabolic cost are modest or inconsistent [53].

In our cohort, the combination of increased walking speed and distance with modest reductions in PCI particularly in the LBG may reflect more stable loading of the paretic limb and decreased cocontraction during stance, leading to a more economical gait pattern at a given workload. This interpretation is consistent with findings of a recent study that showed that exoskeleton‐assisted gait training can enhance lower limb motor function, balance, and walking endurance beyond conventional therapy in chronic stroke [18]. Regarding gait function improvements under E‐KAFO_C, our findings align with studies evaluating powered MPCKAFOs rather than stance–swing devices such as the C‐Brace.

For example, Bulea et al. demonstrated that a powered MPCKAFO prototype (later commercialized as Agilik) improved gait mechanics and energy efficiency in children with cerebral palsy [54]. Similarly, Devine et al. reported functional gains and adaptable torque control in a young adult with spina bifida using Agilik [55], highlighting the potential of powered orthoses for immediate gait assistance.

Our findings align with the seminal work by Lerner et al., who developed a robotic exoskeleton to treat crouch gait in children with cerebral palsy. They reported that powered knee extension assistance during the stance phase improved knee extension by 18.1° while maintaining knee extensor muscle activity [56], indicating that users continued to engage their muscles rather than relying solely on the device. In a subsequent study, Lerner et al. demonstrated that a lower extremity exoskeleton significantly reduced crouch gait during overground walking in the same population. These pediatric findings support our results in adult stroke patients, suggesting that powered knee assistance can provide immediate functional benefits across different neurological conditions while preserving active muscle engagement [57].

The mechanisms underlying these improvements may be similar across populations. In both pediatric cerebral palsy and adult stroke patients, powered orthoses target common gait abnormalities, including insufficient knee extension during stance and impaired knee flexion during swing. The E‐KAFO used in our study, similar to the pediatric exoskeletons developed by Lerner et al., provides targeted assistance that normalizes joint kinematics while preserving neuromuscular activation patterns [56, 57]. In patients with hemiplegia who exhibit a hip hiking or circumduction gait due to foot drop and drag, dorsiflexion assistance from the E‐KAFO likely reduces these compensatory movements, enabling a smoother gait pattern that is more similar to normal walking. Additionally, the knee support feature of the device prevented knee buckling during the early stance phase and helped regulate knee extension during the terminal stance phase, thus addressing common gait abnormalities in patients with stroke, such as genu recurvatum.

Previous studies emphasized the importance of proper knee and ankle control in improving gait function in patients with hemiplegia and stroke [58]. Lee et al. highlighted the critical role of the hip and knee joint coordination in gait function in patients with stroke hemiplegia [59]. Another study reported a significant correlation between changes in plantar pressure and dynamic balance in patients with hemiplegia [60] and the older population [61] classified as having impaired gait, suggesting the importance of ankle control. These studies underscore the necessity of stable knee and ankle control for gait improvement, which is reflected in our findings, where significant improvements in the PCI (indicating energy efficiency) were observed after E‐KAFO application. Previous studies have reported that the use of newly designed KAFOs can enhance gait efficiency in patients with stroke.

In a study by Thijssen et al., the application of a new KAFO in patients with chronic stroke immediately reduced the energy cost of walking (*p* < 0.001) and significantly increased both walking speed and stride length (*p* < 0.005) [62]. Furthermore, after a 3–4‐week adaptation training period, energy consumption was further reduced, indicating enhanced gait efficiency. These findings suggest that wearing an orthosis can improve gait stability and that repetitive training can help patients learn more efficient gait patterns over time.

In this study, significant interaction effects between time and group were observed for specific E‐KAFO_C outcome variables, including the 5‐mWT time, 3‐MWT distance, and 6‐MWT distance. Notably, a significant interaction was found in the 5‐mWT time (*F* = 6.834, *p* = 0.017), with the LBG showing a marked improvement. Furthermore, in the LBG, the application of the E‐KAFO led to a significant reduction in the PCI during the 3‐MWT and 6‐MWT, with decreases of 25.4% (*p* = 0.016) and 19.13% (*p* = 0.025), respectively, indicating a substantial improvement in energy efficiency during gait.

In contrast, the HBG did not show a significant change in the PCI, and the magnitude of the change was smaller than that of the LBG. These results suggest that the assistive functions provided by the E‐KAFO are particularly effective for individuals in the LBG, while they may offer limited benefits for those in the HBG. While the HBG participants already had sufficiently high gait speed and balance control even before the intervention, limiting the extent of further improvement in the LBG participants, who had many limitations in their preintervention gait ability and experienced more substantial benefits from the mechanical support and gait optimization provided by the E‐KAFO [62].

This differential response aligns with observations from pediatric exoskeleton studies, in which powered assistance provided greater benefits to individuals with more pronounced gait impairments [56, 57]. These findings underscore the importance of tailoring gait rehabilitation to a patient’s initial balance and functional capacity and suggest that integrating E‐KAFO use with structured gait training programs may enhance long‐term adaptation and motor learning.

It should be noted that these results were derived from a chronic stroke cohort assessed in a single‐center laboratory under supervised conditions. Consequently, extrapolation to subacute populations, unsupervised community ambulation, or home‐based use should be made with caution. Future multicenter trials encompassing different stages of recovery and real‐world environments are warranted to establish broader external validity.

## 5. Limitations

This study has some limitations.

First, as a pilot study with a small sample size (*n* = 22) and no a priori power calculation, the effect estimates are imprecise, and the generalizability is limited. Moreover, the cohort’s predominantly male composition (19/22) may restrict applicability to female stroke survivors (3/22), as sex‐related differences in anthropometrics and device fit could influence E‐KAFO responsiveness. This sex distribution largely reflects the profile of eligible chronic stroke outpatients attending our rehabilitation center during the recruitment period, rather than a predefined selection preference; however, recruitment bias cannot be completely excluded.

Given potential sex‐related differences in gait pattern and orthotic adaptation, our findings should therefore be interpreted with particular caution when extrapolating to female stroke survivors. Although effect sizes are reported to aid interpretation, the modest sample size means that the study remains underpowered to detect small effects, and nonsignificant findings should therefore be interpreted with caution. These preliminary findings are therefore intended to inform sex‐stratified recruitment, sample size calculation, and design considerations for a future randomized controlled trial (RCT).

Second, postintervention assessments of the BBS, MAS, and MMT were not conducted owing to the limited intervention duration (3 weeks) and substantial assessment burden across two gait conditions. As a result, we were unable to determine whether improvements in gait were accompanied by parallel changes in global balance, spasticity, or muscle strength, and our interpretation of overall functional recovery therefore remains limited. This constrains causal inference and limits our ability to isolate device‐specific effects from nonspecific recovery.

Future studies will incorporate postintervention BBS, MAS, and MMT assessments and will combine these clinical scales with gait and PCI outcomes to provide a more integrated and clinically meaningful interpretation of treatment effect.

Third, this study did not include a conventional rehabilitation‐only control group. Participants were classified according to their ability to balance, and within‐subject comparisons were made between the E‐KAFO_C and BP_C conditions to minimize interindividual variability. A rehabilitation‐only control was not included because all participants were chronic stroke survivors already receiving long‐term therapy and prescribed KAFOs as part of standard care. To more clearly establish the efficacy of the E‐KAFO, future research should adopt a randomized controlled design with a conventional therapy comparator arm.

Fourth, participants continued to receive their usual physiotherapy as part of standard care throughout the study period. Although we asked treating clinicians to keep the rehabilitation regimes largely stable and to avoid introducing new programs specifically for the study, the frequency, content, and intensity of these concurrent sessions were not systematically quantified or analyzed. This uncontrolled concomitant rehabilitation may have contributed to gait improvements independently of the E‐KAFO training, thereby introducing heterogeneity in treatment exposure and potentially confounding the observed effects and affecting internal validity.

Fifth, the intervention duration was limited to 3 weeks, and no follow‐up assessments were performed; thus, the persistence of improvements after training remains unknown. However, given the short‐term nature of these evaluations, interpretations regarding long‐term functional improvements have been made with caution. This constraint reflects the pilot nature of the study, which aimed to simply verify the short‐term efficacy of the E‐KAFO_C and the effectiveness of the BP_C before conducting larger scale trials. Nevertheless, future studies should include extended follow‐up periods (e.g., 3, 6, and 12 months) and multicenter RCTs to assess the durability, functional transfer, and real‐world applicability of observed gains.

Sixth, we did not perform three‐dimensional kinematic or electromyographic analyses of the hemiparetic gait, so our physiological interpretations regarding neuromuscular control and compensatory kinematics remain mostly speculative. Therefore, incorporating three‐dimensional motion capture and muscle activity recordings in future investigations would enable a more comprehensive evaluation of gait quality and compensatory movement patterns; in particular, combining E‐KAFO‐assisted gait training with detailed motion capture and EMG would help clarify how changes in joint kinematics, interlimb coordination, and neuromuscular recruitment patterns relate to improvements in gait performance and PCI.

Finally, because this was a precommercial, exploratory evaluation, the safety and usability monitoring framework was not intended to capture the complete spectrum of potential malfunction or discomfort events. Findings should therefore be interpreted as preliminary signal detection rather than exhaustive surveillance. The absence of a formal adverse event taxonomy or validated usability scales may also have led to underdetection of minor or transient issues. Future trials should preregister a structured safety and usability protocol with standardized definitions and severity grading, include validated instruments (e.g., system usability scale, QUEST, skin integrity, and comfort checklists), and extend exposure duration to characterize the full event profile (see the Safety and Usability Monitoring section).

## 6. Conclusion

In this pilot study, E‐KAFO_C training was associated with short‐term improvements in gait speed, walking distance, and physiological efficiency, with the most pronounced gains observed among participants with lower baseline balance ability. When considered alongside changes observed under the BP_C, these findings provide preliminary evidence that E‐KAFO may enhance functional walking capacity beyond BP orthoses and conventional care.

However, these results should be interpreted as exploratory and hypothesis‐generating rather than definitive proof of efficacy, given the limited sample size, short intervention period, and absence of a rehabilitation‐only control arm. Additionally, future multicenter RCTs with larger samples, longer follow‐up, and comprehensive clinical outcome measures are needed to rigorously evaluate the efficacy of E‐KAFO and the effectiveness of the BP_C, the durability of observed benefits, and the real‐world impact of these approaches in routine rehabilitation practice.

## Author Contributions

Hyuk‐Jae Choi and Mi Jung Kim designed the study. Mi Jung Kim was responsible for participant recruitment and the development of the clinical experimental protocol. Jong‐Won Lee contributed to mechanical design and development of the orthotic system. Yoon Heo was responsible for control algorithms and gait event detection. In Ho Hwang contributed to device certification, testing, and inspection processes. Hyuk‐Jae Choi, Hyeonseok Cho, and Chang‐Yong Ko performed data analysis and interpretation. Ju‐Hwan Bae assisted with manuscript preparation and revision. Chang‐Yong Ko served as the corresponding author and coordinated manuscript submission. The manuscript was drafted by Hyuk‐Jae Choi and Chang‐Yong Ko.

## Funding

This research was supported by the Assistive Technology Commercialize R&D Project for Independent Living for People with Disability and Older People by the Ministry of Health and Welfare, Republic of Korea (Grant Number RS‐2024‐00432986).

## Disclosure

Although one of the authors is affiliated with Refind Inc., this affiliation did not influence the study design, data collection, analysis, interpretation of results, or manuscript preparation. No commercial products were marketed or promoted as part of this research. This declaration is made in accordance with Wiley’s Best Practice Guidelines on Publishing Ethics. All authors reviewed and approved the final manuscript.

## Conflicts of Interest

The authors declare no conflicts of interest.

## Data Availability

Due to ethical restrictions and the protection of personal information, the raw datasets generated and analyzed during the current study are not publicly available. However, deidentified and aggregated data supporting the findings of this study are available from the corresponding author upon reasonable request.
